# Structural characterization of a partially arabinosylated lipoarabinomannan variant isolated from a *Corynebacterium glutamicum ubiA* mutant

**DOI:** 10.1099/mic.0.2007/008078-0

**Published:** 2007-08

**Authors:** Raju Venkata Veera Tatituri, Luke J. Alderwick, Arun K. Mishra, Jerome Nigou, Martine Gilleron, Karin Krumbach, Paul Hitchen, Assunta Giordano, Howard R. Morris, Anne Dell, Lothar Eggeling, Gurdyal S. Besra

**Affiliations:** 1 School of Biosciences, University of Birmingham, Edgbaston, Birmingham B15 2TT, UK; 2 Institut de Pharmacologie et de Biologie Structurale, UMR CNRS 5089, Toulouse, France; 3 Institute for Biotechnology 1, Research Centre Juelich, D-52425 Juelich, Germany; 4 Division of Molecular Biosciences, Faculty of Natural Sciences, Imperial College, London SW7 2AZ, UK; 5 Istituto di Chimica Biomolecolare, Consiglio Nazionale delle Ricerche, 80078 Pozzuoli (NA), Italy; 6 M-Scan Ltd., Wokingham RG41 2TZ, UK

## Abstract

Arabinan polysaccharide side-chains are present in both *Mycobacterium tuberculosis* and *Corynebacterium glutamicum* in the heteropolysaccharide arabinogalactan (AG), and in *M. tuberculosis* in the lipoglycan lipoarabinomannan (LAM). This study shows by quantitative sugar and glycosyl linkage analysis that *C. glutamicum* possesses a much smaller LAM version, Cg-LAM, characterized by single *t*-Ara*f* residues linked to the *α*(1→6)-linked mannan backbone. MALDI-TOF MS showed an average molecular mass of 13 800–15 400 Da for Cg-LAM. The biosynthetic origin of Ara*f* residues found in the extracytoplasmic arabinan domain of AG and LAM is well known to be provided by decaprenyl-monophosphoryl**-**d**-**arabinose (DPA). However, the characterization of LAM in a *C. glutamicum* : : *ubiA* mutant devoid of prenyltransferase activity and devoid of DPA-dependent arabinan deposition into AG revealed partial formation of LAM, albeit with a slightly altered molecular mass. These data suggest that in addition to DPA utilization as an Ara*f* donor, alternative pathways exist in *Corynebacterianeae* for Ara*f* delivery, possibly via an unknown sugar nucleotide.

## INTRODUCTION

The *Corynebacterianeae* represent a distinct and unusual group within Gram-positive bacteria, with the most prominent members being the human pathogens *Mycobacterium tuberculosis* and *Mycobacterium leprae* (Bloom & Murray, 1992). Non-pathogenic bacteria also belong to this taxon, such as *Corynebacterium glutamicum*, which is used in the industrial production of amino acids (Sahm *et al.*, 2000) . These bacteria collectively belong to the same suborder, and share a similar genome, cell envelope and corresponding cell wall biosynthetic enzymes (Dover *et al.*, 2004; Stackebrandt *et al.*, 1997).

The characteristic cell envelope of this distinct group of bacteria contains mycolic acids, arabinogalactan (AG) and peptidoglycan, which are covalently linked to each other to form the mycolyl-arabinogalactan-peptidoglycan (mAGP) complex (Besra *et al.*, 1995; Brennan & Nikaido, 1995; Brennan, 2003; Daffé *et al.*, 1990; Dmitriev *et al.*, 2000; Dover *et al.*, 2004; McNeil *et al.*, 1990, 1991). In addition, they possess an array of cell-wall-associated glycolipids, such as phosphatidyl-*myo*-inositol (PI), phosphatidyl-*myo*-inositol mannosides (PIMs) and the lipoglycans lipomannan (LM) and lipoarabinomannan (LAM) (Brennan & Ballou, 1967, 1968; Chatterjee *et al.*, 1991, 1993; Guerardel *et al.*, 2002; Hill & Ballou, 1966; Khoo *et al.*, 1995). As a result, arabinose is present in two polysaccharides with markedly different structures. The occurrence of arabinosyl-containing glycoconjugates in bacteria (Brennan & Nikaido, 1995), plants (Fincher *et al.*, 1983) and protozoan parasites (Dobson *et al.*, 2003; Goswami *et al.*, 2006; Guha-Niyogi *et al.*, 2001; Previato *et al.*, 1982; Xavier Da Silveira *et al.*, 1998) and their absence in mammalian cells is well known. *Corynebacterianeae* arabinan is an extremely complex homopolymer of d-arabinofuranose (d-Ara*f*) residues based on discrete structural motifs; however, the complete structures and biosynthesis of these polymers are still to be established.

For LAM biosynthesis we initially proposed the following biosynthetic pathway PI→PIM→LM→LAM (Besra *et al.*, 1997) in *M. tuberculosis*, which is now largely supported by biochemical and genetic evidence (Gurcha *et al.*, 2002; Kordulakova *et al.*, 2002; Kremer *et al.*, 2002; Schaeffer *et al.*, 1999). PimA catalyses the addition of Man*p* provided by GDP-mannose to the 2-position of the *myo*-inositol of PI to form PIM_1_ (Kordulakova *et al.*, 2002), whereas PimB might be responsible for the addition of a second Man*p* to the 6-position to yield Ac_1_PIM_2_ (Schaeffer *et al.*, 1999; Tatituri *et al.*, 2007). PimC has been demonstrated to allow further mannosylation to Ac_1_PIM_3_ (Kremer *et al.*, 2002) and more recently, PimE has been shown to be involved in the biosynthesis of Ac_1_PIM_5_ (Morita *et al.*, 2006). It has been proposed that PIM_4_ is the direct precursor of LM, characterized by a linear *α*(1→6)-linked mannan backbone linked with *α*(1→2) mannopyranose side chains generated through a mannosyltransferase encoded by Rv2181 (Kaur *et al.*, 2006). LM is then further glycosylated with arabinan to produce LAM, and finally ‘mannose-capped’ to produce ManLAM, a process initiated by the capping enzyme encoded by Rv1635c (Dinadayala *et al.*, 2006). For the addition of arabinose residues into mycobacterial LAM, EmbC is required (Berg *et al.*, 2005; Shi *et al.*, 2006; Zhang *et al.*, 2003), whereas AftA, EmbA and EmbB perform arabinan polymerization in AG (Alderwick *et al.*, 2006b; Belanger *et al.*, 1996; Escuyer *et al.*, 2001). In contrast, *C. glutamicum* possesses a singular Emb, and an AftA orthologue, which are used in AG biosynthesis (Alderwick *et al.*, 2005, 2006b; Seidel *et al.*, 2007). It is interesting to note that the arabinan domains of AG and LAM utilize several different Ara*f* linkages, which suggests that additional arabinofuranosyltransferases must be required to form AG and LAM, and still remain to be identified in *Corynebacterianeae*.

It has been shown that the activated Ara*f* sugar donor in *Corynebacterianeae* is decaprenyl-monophosphoryl**-**d**-**arabinose (DPA) (Wolucka *et al.*, 1994). It was also proposed that decaprenyl-monophosphoryl**-**d**-**ribose (DPR) could be an additional precursor involved in arabinan synthesis, via a 2′-epimerase (Wolucka & de Hoffmann, 1995). Recently, it was shown that 5-phosphoribofuranose pyrophosphate (pRpp) is converted to decaprenyl-monophosphoryl-5-phosphoribose (DPPR) via a 5-phospho-*α***-**d**-**ribose-1-diphosphate-decaprenyl-phosphate 5-phosphoribosyltransferase (UbiA; Rv3806c and NCgl2781 in *M. tuberculosis* and *C. glutamicum*, respectively) (Huang *et al.*, 2005), which is then dephosphorylated to form DPR. DPR is then enzymically oxidized to form a keto-intermediate, DPX, followed by an enzymic reduction to DPA (Mikusova *et al.*, 2005). Additionally, disruption in *C. glutamicum* of *ubiA*, encoding the first enzyme involved in the biosynthesis of DPA, resulted in a complete loss of cell wall arabinan (Alderwick *et al.*, 2006a). To date, no sugar nucleotides of Ara*f* have been identified. The key discovery that Ara*f* residues in AG arise from DPA also raised the question whether mycobacteria neither produce nor require Ara*f* sugar nucleotides. Herein, we now clearly show that an *ubiA*-disrupted mutant of *C. glutamicum* still produces a modified LAM variant (Cg-LAM), which is arabinosylated even in the absence of the sugar donor DPA (Alderwick *et al.*, 2006a). Taken together, the data suggest that alternative pathways may exist in *Corynebacterianeae*, independent of DPA utilization, specifically involved in arabinosylation events in Cg-LAM biosynthesis.

## METHODS

### Strains, construction of plasmids and culture conditions.

*C. glutamicum* ATCC 13032 (the wild-type strain, referred to for the remainder of the text as *C. glutamicum*) and *Escherichia coli* DH5*α*mcr were grown in Luria–Bertani broth (Difco) at 30 °C and 37 °C, respectively. The *C. glutamicum* : : *ubiA m*utant was grown on the complex medium brain heart infusion (Difco). Kanamycin and ampicillin were used at a concentration of 50 μg ml^−1^. The vector used for inactivation of *C*. *glutamicum* : : *ubiA* was pCg : : *ubiA*. It contained an internal *ubiA* fragment of 321 bp amplified with the primer pairs ATC TTC AAC CAG CGC ACG ATC and AAT ATC GAT CAC TGG CAT GTG, which was ligated into the *Sma*I site of the non-replicative vector pK18mob to yield pCg : : *ubiA*. To enable chromosomal inactivation of *ubiA*, pCg : : *ubiA* was introduced into *C. glutamicum* by electroporation (Alderwick *et al.*, 2005).

### Extraction and purification of lipoglycans.

Purification procedures were adapted from protocols established for the extraction and purification of mycobacterial lipoglycans (Nigou *et al.*, 1999). Briefly, 10 g of cells grown to an OD_600_ of 1.0, were delipidated at 60 °C by using 500 ml CHCl_3_/CH_3_OH (1 : 1, v/v). The delipidated cells were resuspended in deionized water and disrupted by probe sonication (MSE Soniprep 150, 12 μm amplitude, 60 s on, 90 s off for 10 cycles, on ice) and the cell debris refluxed five times with 100 ml C_2_H_5_OH/H_2_O (1 : 1, v/v) at 68 °C, for 12 h intervals. The cell debris was removed by centrifugation and the supernatant containing lipoglycans, neutral glycans and proteins dried. The dried supernatant was then treated with a hot 80 % (w/w) phenol/H_2_O biphasic wash at 70 °C for 1 h, followed by several protease treatments, and the lipoglycan fraction was recovered following extensive dialysis against deionized water (Nigou *et al.*, 1999).

The crude lipoglycan extract was resuspended in buffer A (50 mM ammonim acetate and 15 % propan-1-ol) and subjected to octyl-Sepharose CL-4B hydrophobic interaction chromatography (2.5 cm×50 cm) as previously reported (Leopold & Fischer, 1993). The column was initially washed with 4 column volumes of buffer A to ensure removal of neutral glycans followed by an increasing gradient of propan-1-ol ranging from 25 to 65 %, keeping the concentration of ammonium acetate constant. The eluates were collected and extensively dialysed against deionized water, concentrated to approximately 1 ml and precipitated using 5 ml C_2_H_5_OH; the sample was freeze-dried using a Savant SpeedVac. The freeze-dried sample containing the retained material from the hydrophobic interaction column was then resuspended in buffer B (0.2 M NaCl, 0.25 %, w/v, sodium deoxycholate, 1 mM EDTA and 10 mM Tris/HCl, pH 8) to a final concentration of 200 mg ml^−1^. The sample was gently mixed and left to incubate for 48 h at room temperature. The sample was then loaded onto a Sephacryl S-200 column (2.5 cm×50 cm) previously equilibrated with buffer B. The sample was washed with 400 ml buffer C (0.2 M NaCl, 1 mM EDTA and 10 mM Tris/HCl, pH 8) at a flow rate of 3 ml h^−1^, collecting 1.5 ml fractions using a Bio-Rad auto-sampler. The fractions were monitored by SDS-PAGE using either a silver stain utilizing periodic acid and silver nitrate (Hunter *et al.*, 1986) or a Pro-Q emerald glycoprotein stain (Invitrogen), and individual fractions were pooled and dialysed extensively against buffer C for 72 h with frequent changes. The samples were further dialysed against deionized water for 48 h with frequent changes of deionized water, lyophilized and stored at −20 °C.

### Glycosyl composition and linkage analysis of lipoglycans by alditol acetates.

Lipoglycans from wild-type *C. glutamicum* and the *C. glutamicum* : : *ubiA* mutant were hydrolysed using 2 M trifluoroacetic acid (TFA), reduced with NaBD_4_, and the resultant alditols were per-*O*-acetylated and examined by GC (Tatituri *et al.*, 2007). Lipoglycans (2 mg) were per-*O*-methylated using dimethyl sulfinyl carbanion as described previously (Alderwick *et al.*, 2005; Besra *et al.*, 1995; Daffé *et al.*, 1990). In this procedure, lipoglycan samples (2 mg) were resuspended in 0.5 ml DMSO (anhydrous) and 100 μl of 4.8 M dimethyl sulfinyl carbanion. The reaction mixture was stirred for 1 h and then iodomethane (50 μl) was slowly added and the suspension stirred for 1 h; this process was repeated for a total of three times. The reaction mixture was then diluted with an equal volume of water, dialysed against deionized water and dried. The resulting retentate was applied to a pre-equilibrated C_18_ Sep-Pak cartridge and initially washed with 10 ml water, 10 ml 20 % acetonitrile, and the per-*O*-methylated lipoglycan eluted with 2 ml acetonitrile and 2 ml ethanol. After drying the combined organic eluate under nitrogen, the per-*O*-methylated lipoglycan was hydrolysed using 250 μl 2 M TFA at 120 °C for 2 h. The resulting hydrolysate was reduced with NaBD_4_, per-*O*-acetylated and examined by GC/MS (Tatituri *et al.*, 2007). Per-*O*-methylation of lipoglycans for matrix-assisted laser desorption ionization-mass spectrometry (MALDI-MS) analysis was performed as described previously (Dell *et al.*, 1993). Briefly, 1 ml of a DMSO/NaOH slurry was added followed by 0.5 ml of iodomethane. The reaction mixture was vigorously shaken for 10 min at room temperature and the reaction quenched with 1 ml water. Per-*O*-methylated samples were then extracted into chloroform (1 ml) and washed several times with water before drying under a stream of nitrogen.

### GC and GC/MS analysis.

Analysis of partially per-*O*-methylated, per-*O*-acetylated alditol acetate sugar derivatives was performed on a CE Instruments ThermoQuest Trace GC 2000. Samples were injected in the split mode. The column used was a DB225 (Supelco). The oven was programmed to hold at an isothermal temperature of 275 °C for a run time of 15 min. GC/MS was carried out on a BPX5 column (Supelco) and a Finnigan Polaris/GCQ PlusTM, as described previously (Besra *et al.*, 1995; Daffé *et al.*, 1990). GC/MS analysis of alditol acetate sugar derivatives was performed on a Perkin Elmer Clarus 500 instrument. Samples were injected in the splitless mode. The column used was an RTX-5 (30 m×0.25 mm internal diameter, Restek Corp.). Initial temperature was set at 60 °C then ramped to 300 °C at 8 °C  min^−1^.

### NMR spectroscopy.

NMR spectra of lipoglycans were recorded on a Bruker DMX-500 equipped with a double-resonance (1H/X)-BBi *z*-gradient probe head. All samples were exchanged in D_2_O (D, 99.97 %; Euriso-top), with intermediate lyophilization, and then dissolved in 0.5 ml D_2_O and analysed at 313 K. The ^1^H and ^13^C NMR chemical shifts were referenced relative to internal acetone at 2.225 and 34.00 p.p.m., respectively. All the details concerning NMR sequences used and experimental procedures were described in previous studies (Gilleron *et al.*, 1999, 2000; Nigou *et al.*, 1999).

### MALDI-MS analysis.

MALDI-MS was performed using a PerSeptive Biosystems Voyager DE STR mass spectrometer. Native lipogycans were dissolved in CH_3_OH/H_2_O (50 : 50, v/v) and 1 μl of the sample was loaded onto a metal plate. After evaporation, 1 μl of the matrix 2,5-dihydroxybenzoic acid was added on the spot. Samples were analysed using the linear negative mode. Per-*O*-methylated samples were dissolved in CH_3_OH/H_2_O (80 : 20, v/v) and analysed in the linear positive mode using 2,5-dihydroxybenzoic acid as matrix. Sequazyme peptide mass standards (Applied Biosystems) were used as external calibrants.

## RESULTS

### Disruption of *Cg-ubiA*

The *ubiA* gene product was shown in prior work to synthesize DPPR, which is converted to DPA, thus supplying the substrate for the ‘priming’ arabinosyltransferase AftA (Alderwick *et al.*, 2006b). These initial Ara*f* residues ‘prime’ the galactan backbone for further attachment of *α*(1→5)-linked Ara*f* residues. These reactions require the arabinofuranosyltransferase activities of Mt-EmbA and Mt-EmbB, or Cg-Emb, respectively, and also the utilization of DPA (Alderwick *et al.*, 2005; Escuyer *et al.*, 2001; Seidel *et al.*, 2007), to eventually result in mature AG. In light of our present studies demonstrating the occurrence of Cg-LAM we inactivated the mycobacterial orthologue of *C. glutamicum*, NCgl2781, by transforming the wild-type to kanamycin resistance conferred by the vector-borne *aph* gene product of pCg : : *ubiA* with a view to generating a clear *C. glutamicum* LM-only phenotype. The vector, as previously described (Alderwick *et al.*, 2005), was integrated into the chromosomal *ubiA* gene, thus disrupting *ubiA*, as confirmed by two independent PCR analyses with two different primer pairs (Alderwick *et al.*, 2005). As expected, the resulting strain *C. glutamicum* : : *ubiA* exhibited strongly reduced growth (Alderwick *et al.*, 2006a).

### Purification of lipoglycans from *C. glutamicum* and *C. glutamicum* : : *ubiA*

**Fig. 1. f1:**
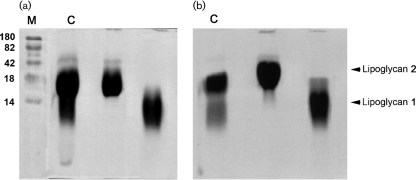
Lipoglycan profiles of wild-type *C. glutamicum* (a) and *C. glutamicum* : : *ubiA* (b). Lipoglycans were analysed using SDS-PAGE and visualized using the Pro-Q emerald glycoprotein stain (Invitrogen) specific for carbohydrates. Individual lipoglycans (1 and 2) were purified as described in Methods. C, crude lipoglycan; M, molecular mass markers (kDa).

Analysis of the crude phenol-extracted lipoglycans from *C. glutamicum* (Fig. 1a[Fig f1]) and *C. glutamicum* : : *ubiA* (Fig. 1b[Fig f1]) visualized on SDS-PAGE revealed two closely migrating lipoglycans. A two-step purification protocol was performed to fractionate the crude lipoglycans. In the first step, the neutral glycans and nucleic acids were eliminated by using an octyl-Sepharose CL-4B column (Amersham Biosciences). In the second step, the lipoglycans were individually purified using a Sephacryl S-200 column (Amersham Biosciences). Fractions containing the lipoglycans were monitored by SDS-PAGE stained with either silver nitrate or Pro-Q emerald glycoprotein stains and pooled accordingly to afford lower (1) and upper (2) lipoglycans (Fig. 1a, b[Fig f1]).

### General structural features of lipoglycans from *C. glutamicum* and *C. glutamicum* : : *ubiA*

**Fig. 2. f2:**
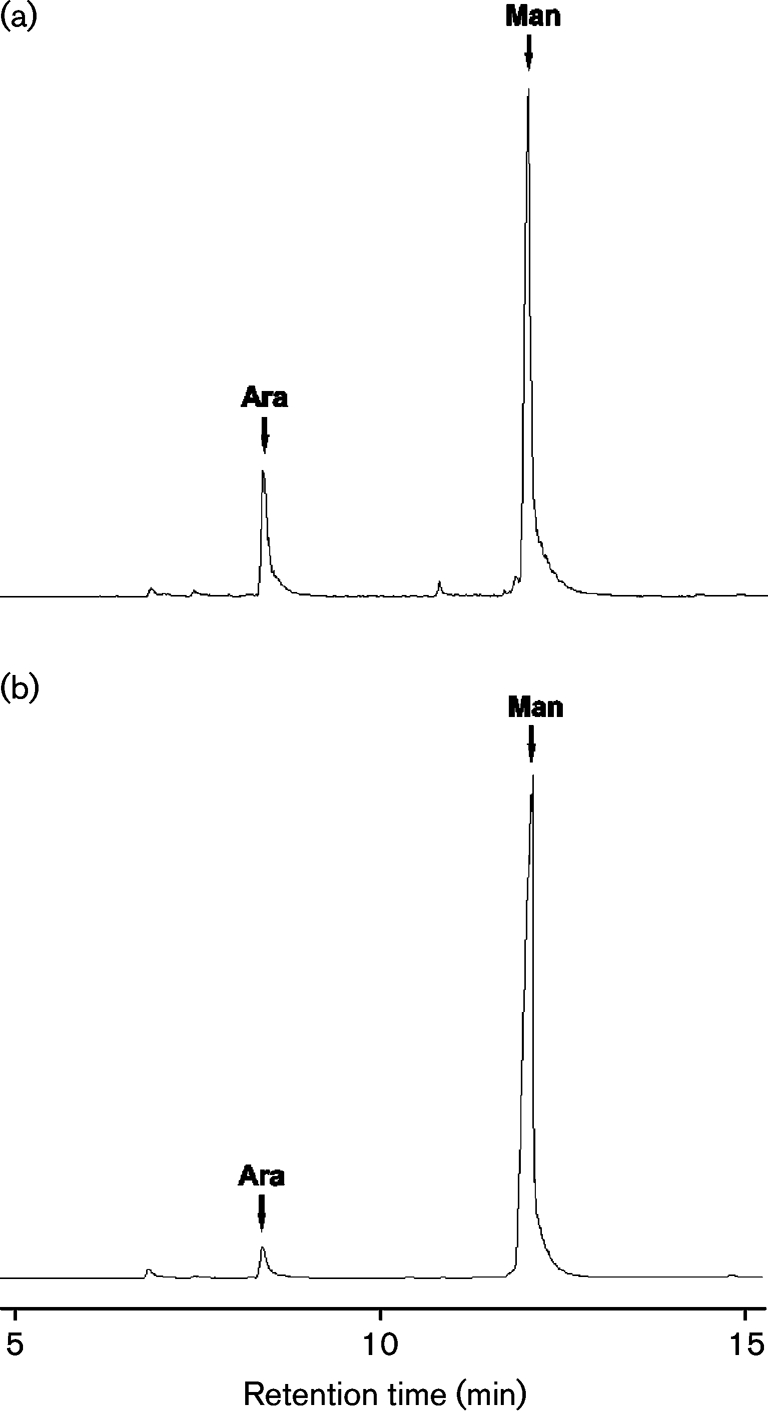
Glycosyl compositional analysis of lipoglycan-2 from wild-type *C. glutamicum* (a) and *C. glutamicum* : : *ubiA* (b). Samples of individually purified lipoglycans (2) were hydrolysed with 2 M TFA, reduced and per-*O*-acetylated. Alditol acetates were subjected to GC analysis. Peak area integration shows that the arabinose content is 23 % in wild-type *C. glutamicum* (a) and 4.5 % in the *C. glutamicum* : : *ubiA* mutant (b). Ara, arabinose; Man, mannose.

**Fig. 3. f3:**
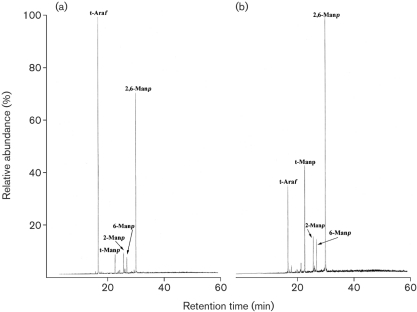
Glycosyl linkage analysis of per-*O*-methylated lipoglycan-2 from *C. glutamicum* (a) *and C. glutamicum* : : *ubiA* (b). Lipoglycan 2 from *C. glutamicum* and *C. glutamicum* : : *ubiA* was per-*O*-methylated, hydrolysed using 2 M TFA, reduced, and per-*O*-acetylated. The resulting partially per-*O*-methylated and per-*O*-acetylated glycosyl derivatives were analysed by GC/MS as described previously (Alderwick *et al.*, 2005).

**Fig. 4. f4:**
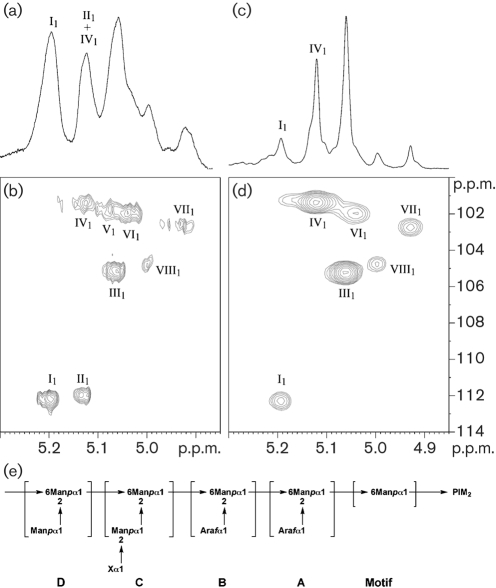
(a–d) NMR characterization of wild-type Cg-LAM (a, b) and Cg-LAM from the UbiA mutant (c, d). 1D ^1^H (a, c) and 2D ^1^H-^13^C HMQC (b, d) NMR spectra of Cg-LAMs in D_2_O at 313 K. Expanded regions (*δ* ^1^H: 4.85–5.30) (a, c) and (*δ* ^1^H: 4.85–5.30, *δ* ^13^C: 100–114) (b, d) are shown. Glycosyl residues are labelled in Roman numerals and their carbons and protons in Arabic. I, II, *t*-*α*-Ara*f*; III, *t*-*α*-Man*p*; IV, V, VI, 2,6-*α*-Man*p*; VII, 6-*α*-Man*p*; VIII, 2-*α*-Man*p*. (e) Structural representation of Cg-LAM (Tatituri *et al.*, 2007). Cg-LAM contains a (1→6)-Man*p* backbone almost completely substituted by *t*-Ara*f*, *t*-Man*p*, *t*-Man*p*(1→2)-Man*p* and t-Ara*f*(1→2)-Man*p* units. X, either a *t*-Ara*f* or a *t*-Man*p* unit.

The lower lipoglycans (1) from both *C. glutamicum* and *C. glutamicum* : : *ubiA* were similar and exhibited the basic components of a structure related to mycobacterial LM (now termed Cg-LM) and contained solely mannopyranose (Man*p*). Per-*O*-methylation analysis of Cg-LM from both strains indicated the presence of *t*-Man*p*, 2-Man*p*, 6-Man*p* and 2,6-Man*p* residues (Tatituri *et al.*, 2007). GC analysis of the total acid-hydrolysed upper lipoglycan (2) from *C. glutamicum* showed that it contained arabinose and mannose in a ratio of 23 : 77 and was related to mycobacterial LAM (Fig. 2a[Fig f2]). Per-*O*-methylation analysis of wild-type Cg-LAM indicated the presence of *t*-Ara*f*, *t*-Man*p*, 2-Man*p*, 6-Man*p* and 2,6-Man*p* residues (Fig. 3a[Fig f3]). Interestingly, Cg-LAM of the wild-type strain was previously shown to be composed of a PI anchor linked to an *α*(1→6)Manp backbone substituted at most of the *O*-2 positions by the structural motifs *t*-Ara*f*, *t*-Man*p*, *t*-Ara*f*(1→2)-Man*p* and *t*-Man*p*(1→2)-Man*p* units (Fig. 4e[Fig f4]) (Tatituri *et al.*, 2007). Accordingly, the 1D ^1^H-NMR anomeric region (Fig. 4a[Fig f4]) exhibited a complex pattern of overlapping resonances corresponding to Ara*f* and Man*p* units. The different spin systems were characterized by ^1^H-^13^C HMQC NMR (Fig. 4b[Fig f4]) and anomeric resonances were attributed as follows: *δ*H_1_C_1_5.20/112.2 (I_1_) to two overlapping *t*-Ara*f* units (see below) and 5.13/112.0 (II_1_) to a third *t*-Ara*f* unit, 5.06/105.2 (III_1_) to *t*-Man*p* units, 5.12/101.4 (IV_1_), 5.07/101.7 (V_1_) and 5.04/101.9 (VI_1_) to 2,6-Man*p* units, 5.06/105.2 (VII_1_) to 6-Man*p* units, and 5.00/104.9 (VIII_1_) to 2-Man*p* units.

Our previous experiments demonstrated that the *C. glutamicum* : : *ubiA* mutant failed to synthesize DPA (Alderwick *et al.*, 2006a) and possessed an arabinan-deficient cell wall phenotype (Alderwick *et al.*, 2005). As a consequence, we anticipated that the Cg-LAM from the mutant would be devoid of arabinose. Surprisingly, glycosyl compositional analysis of Cg-LAM from the *C. glutamicum* : : *ubiA* mutant showed that it contained arabinose, albeit in substantially smaller amounts, and mannose in a ratio of 4.5 : 95.5 (Fig. 2b[Fig f2]). Per-*O*-methylation analysis of Cg-LAM from the mutant indicated the presence of *t*-Ara*f*, *t*-Man*p*, 2-Man*p*, 6-Man*p* and 2,6-Man*p*, although the relative abundance of *t*-Ara*f* residues was reduced (Fig. 3b[Fig f3]). In agreement with the glycosyl compositional and linkage analysis data indicating a reduced amount of *t*-Ara*f* units, the 1D ^1^H anomeric region of Cg-LAM from the *glutamicum* : : *ubiA* mutant (Fig. 4c[Fig f4]) was simpler than that observed for Cg-LAM from wild-type *C. glutamicum* (Fig. 4a[Fig f4]). Resonance I_1_, corresponding to a *t*-*α*-Ara*f* unit, was dramatically reduced, suggesting two overlapping *t*-Ara*f* signals. This was further confirmed by ^1^H-^13^C HMQC analysis (Fig. 4d[Fig f4]). Indeed, the relative intensity of the correlation at *δ*H_1_C_1_5.20/112.2 (I_1_) was much weaker and correlation at *δ*H_1_C_1_ 5.13/112.0 (II_1_) of the third *t*-Ara*f* residue was absent.

**Fig. 5. f5:**
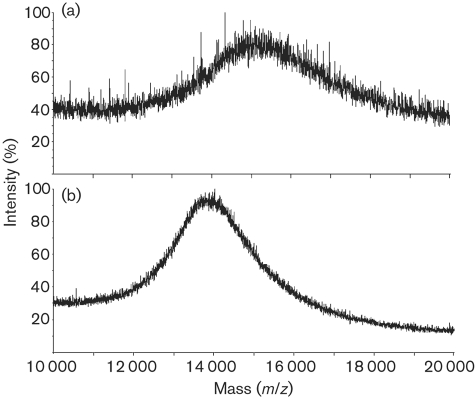
MALDI-TOF MS spectra of Cg-LAM from wild-type *C. glutamicum* (a) and *C. glutamicum* : : *ubiA* (b). MALDI spectra were acquired in the linear negative mode with delayed extraction using 2,5-dihydrobenzoic acid as matrix.

The native Cg-LAMs from wild-type *C. glutamicum* and the *C. glutamicum* : : *ubiA* mutant were analysed by MALDI TOF MS. MALDI spectra were performed in the linear negative mode. The mass spectra show broad unresolved molecular ion envelopes due to the heterogeneity of the Cg-LAMs and the relatively low resolution of this type of MS. The native Cg-LAM from wild-type *C. glutamicum* shows an average molecular mass of around 15 400 Da (Fig. 5a[Fig f5]). The native Cg-LAM from the *C. glutamicum* : : *ubiA* mutant shows a somewhat lower average molecular mass in the region of 13 900 Da (Fig. 5b[Fig f5]). Samples of Cg-LAM from wild-type *C. glutamicum* and *C. glutamicum* : : *ubiA* were subjected to per-*O*-methylation and analysed by MALDI TOF MS (data not shown). Per-*O*-methylation replaces fatty acyl substituents with methyl groups as well as methylating all the free hydroxyl groups. The average molecular mass of the derivatized Cg-LAM from wild-type *C. glutamicum* was observed around 15 600 Da, whilst the molecular mass of the corresponding Cg-LAM from *C. glutamicum : : ubiA* was observed around 15 200 Da.

Altogether, these data indicate that the *C. glutamicum* : : *ubiA* mutant produces a slightly truncated Cg-LAM with a severe reduction in *t*-Ara*f* content but maintaining and possibly extending its mannan core. In addition, the presence of UbiA and the biosynthesis of DPA appear to be essential for the higher arabinosylation of Cg-LAM. Moreover, these findings suggest that arabinose deposition in the *C. glutamicum* : : *ubiA* mutant is a result of incorporation of *t*-Ara*f* through another arabinose substrate, and one which is not DPA dependent.

## DISCUSSION

Understanding the biosynthesis of mycobacterial arabinan is paramount to identifying potential new drug targets for the treatment of tuberculosis. However, as discussed earlier our understanding of these pathways is far from complete. In this respect, *C*. *glutamicum* is a useful tool in understanding mycobacterial cell wall biosynthesis, since this organism possesses the core structural elements of *Corynebacterianeae* with few gene duplications, and deletion of orthologous genes, which is often lethal in mycobacterial species, is possible (Alderwick *et al.*, 2005, 2006b; Gande *et al.*, 2004). Specifically, the deletion of the DPA requiring arabinosyltransferase Emb in *C. glutamicum* resulted in a strongly abrogated arabinan domain of AG (Alderwick *et al.*, 2005), and deletion of AftA also disabled attachment of the remaining singular *t*-Ara*f* residues to galactan (Alderwick *et al.*, 2006b). Furthermore, the loss of DPA synthesis in a *C. glutamicum* : : *ubiA* mutant also resulted in the full loss of the arabinan domain in mature AG (Alderwick *et al.*, 2005). In contrast, Cg-LM and Cg-LAM isolated from the corresponding *C. glutamicum* Δ*emb* and Δ*aftA* mutants were similar to the wild-type glycans (data not shown). An absence of Ara*f* residues was expected in Cg-LAM of the *C. glutamicum* : : *ubiA* mutant. Strikingly, a total absence of arabinan was not observed (Fig. 2), thus requiring an alternative source and mechanism of addition of this sugar which may be attributed to the presence of an uncharacterized nucleotide sugar donor in *Corynebacterineae*.

In contrast to mycobacteria, parasites do synthesize and utilize a sugar nucleotide to provide an activated arabinose sugar donor. Schneider *et al.* (1994) characterized GDP-*α***-**d**-**Ara*p*, the precursor of d-Ara in *Leishmania major* lipophosphoglycan. In trypanosomatids, this sugar nucleotide is believed to be synthesized in a two-step process through the combined activities of an Ara-1-kinase and GDP-Ara pyrophosphorylase, whereby arabinose is phosphorylated to form arabinose 1-phosphate and then activated to the nucleotide level by GDP**-**d**-**Ara*p* pyrophosphorylase (Mengeling & Turco, 1999; Schneider *et al.*, 1995). Compared with the many genes encoding glycosyltransferases in prokaryotic and eukaryotic systems, very little is known about those that encode arabinopyranosyltransferases. Dobson *et al.* (2003) identified two genes (*SCA1/2*) encoding arabinosyltransferases mediating scAra capping, and recently it was demonstrated by heterologous expression that *Leishmania SCA1* encodes an arabinopyranosyltransferase (Goswami *et al.*, 2006).

Glycosyltransferases belonging to one of the families, GT-A and GT-B, or both, may be responsible for the initial addition of *t*-Ara*f* residues on to Cg-LAM in the absence of DPA. These enymes may be present on the cytoplasmic side of the membrane and probably aid in the transfer of *t*-Ara*f* residues from a putative sugar nucleotide donor. These results could explain the presence of *t*-Ara*f* in Cg-LAM, even though DPA biosynthesis has been completely abrogated in the *C*. *glutamicum* : : *ubiA* mutant (Alderwick *et al.*, 2005). The mechanism by which these Ara*f* residues are added is not clear. One possible explanation is that Ara*f* residues from a nucleotide precursor are added in a step-wise fashion to Cg-LM on the cytoplasmic side to give motif A proximal to PI, and then this complex polysaccharide is then probably flipped on to the periplasmic side. It is thought that the flipping mechanism is a prerequisite for arabinosylation to occur outside of the membrane, where the arabinan and mannan domains are formed through specific GT-C glycosyltransferases, by the addition of either *t-*Ara*f* and *t*-Man*p* residues, from DPA or decaprenyl-monophosphoryl**-**d**-**mannose (DPM) to give motifs B, C and D (Figs 4e[Fig f4] and 6) (Berg *et al.*, 2007; Seidel *et al.*, 2007; Tatituri *et al.*, 2007). The biosynthetic origin of mycobacterial Ara*f* residues is generally a poorly understood area. To date, no sugar nucleotides of Ara*f* have been identified, and as a result, a number of theories regarding the generation of arabinan in mycobacteria have been put forward (Huang *et al.*, 2005; Mikusova *et al.*, 2005; Scherman *et al.*, 1995, 1996). Depending on the organization of the arabinan chains, it is speculated that five or six arabinofuranosyltransferases are required for arabinan biosynthesis in mycobacteria. In this regard, it is interesting to note that an *M. smegmatis embC* mutant (Escuyer *et al.*, 2001; Zhang *et al.*, 2003) was found to be devoid of LAM but from the chemical analysis, the resulting LM precursor possessed two or three Ara*f* residues. The possibility exists that these residues are initially added via a non-DPA-dependent glycosyltransferase; this requires further investigation.

## Figures and Tables

**Fig. 6. f6:**
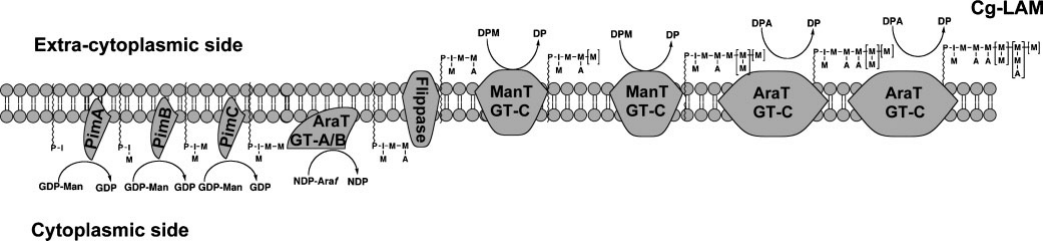
Proposed biosynthetic pathway of Cg-LAM. The addition of the first Ara*f* unit is thought to occur on the cytoplasmic side of the membrane by a GT-A/B glycosyltransferase (Liu & Mushegian, 2003). After transportation across the membrane by an unknown ‘flippase’ enzyme, further elaboration of the lipoglycan then occurs through the addition of mannose and arabinose units catalysed by several GT-C glycosyltransferases utilizing DPM and DPA, respectively (Berg *et al.*, 2007; Seidel *et al.*, 2007; Tatituri *et al.*, 2007).

## Abbreviations

- Ac, acylated
- Ara, arabinose
- Ara*f*, arabinofuranose
- AG, arabinogalactan
- DPA, decaprenyl-monophosphoryl**-**d**-**arabinose
- DPM, decaprenyl-monophosphoryl-mannose
- DPR, decaprenyl-monophosphoryl**-**d**-**ribose
- DPPR, decaprenyl-monophosphoryl-5-phosphoribose
- LAM, lipoarabinomannan
- LM, lipomannan
- Man, mannose
- mAGP, mycolyl-arabinogalactan-peptidoglycan
- *p*, pyranose
- PI, phosphatidyl-*myo*-inositol
- PIMs, phosphatidyl-*myo*-inositol mannosides
- pRpp, 5-phosphoribofuranose pyrophosphate
- *t*-, terminal
- TFA, trifluoroacetic acid
